# Extended endoscopic transorbital approach for mesial temporal lobe glioma

**DOI:** 10.3171/2025.1.FOCVID24189

**Published:** 2025-04-01

**Authors:** Chiman Jeon, Chang-Ki Hong

**Affiliations:** 1 Department of Neurosurgery, Korea University Ansan Hospital, Korea University College of Medicine, Ansan, Gyeonggi-do; and; 2 Department of Neurosurgery, Asan Medical Center, University of Ulsan College of Medicine, Seoul, Republic of Korea

**Keywords:** extended endoscopic transorbital approach, high-lying eyelid incision, lateral orbitotomy, mesial temporal lobe, glioma

## Abstract

The endoscopic transorbital approach (ETOA) is a minimally invasive technique that provides direct access to the mesial temporal lobe while avoiding brain retraction, preserving the lateral temporal neocortex, and minimizing the risk of damage to the optic radiations. In this video, the authors demonstrate an extended ETOA with lateral orbitotomy for the resection of a mesial temporal lobe glioma, emphasizing its utility along the long axis of the amygdala-hippocampal complex. This video is intended to serve as a practical guide for accessing the mesial temporal lobe through the extended transorbital corridor.

The video can be found here: https://stream.cadmore.media/r10.3171/2025.1.FOCVID24189

## Transcript

In this video, we demonstrate an extended endoscopic transorbital approach for resection of mesial temporal lobe glioma.

### 0:28 Case Presentation and Preoperative Imaging.

A 45-year-old male presented with chronic headache. Neurological examination was unremarkable. Preoperative imaging revealed a diffusely infiltrating lesion involving the mesial temporal lobe.

### 0:41 Case Presentation and Preoperative Imaging.

On the coronal images, you can see the tumor sitting medial to the temporal horn.

### 0:47 Anatomy of Mesial Temporal Lobe.

Anatomically, the mesial temporal lobe can be divided into three parts: anterior, middle, and posterior.^1^ In this case, the infiltrating lesion appears to involve the anterior and middle segments of the mesial temporal lobe.

### 1:03 Surgical Approaches for Mesial Temporal Lobe Glioma.

So, for this deep-seated lesion, we can consider six surgical approaches.

### 1:08 Conventional Transcranial Approaches.

Firstly, there are three conventional transcranial approaches for accessing the mesial temporal lobe: transsylvian, transtemporal, and subtemporal.^2^

### 1:20 Conventional Transcranial Approaches.

And each approach has its respective advantages and pitfalls.^2,3^ They are particularly useful when the tumor involves the anterior and middle segments of the mesial temporal lobe, as in our case. However, they often come with the risk of damaging the optic radiations and require some degree of brain retraction.

### 1:41 Supracerebellar Transtentorial Approach.

In contrast, when a target lesion involves the middle and posterior segments, especially medial to the collateral sulcus, the supracerebellar transtentorial approach can be considered.^4^

### 1:53 Endoscopic Transorbital Approach.

On the other hand, the endoscopic transorbital approach offers direct access to the mesial temporal lobe without brain retraction, thereby sparing the lateral neocortex.^5^ It also obviates the need for a standard frontotemporal craniotomy. This minimally invasive approach provides an anterior-to-posterior route that is inferomedial to the optic radiations, thereby reducing the risk of injury to the nearby optic pathway.

### 2:21 Levels of Difficulty for Transorbital Access to Mesial Temporal Lobe.

In addition, this transorbital access to the mesial temporal lobe corresponds to stage 4 according to the levels of difficulty for endoscopic transorbital surgery.^6^

### 2:32 Modified Lateral Orbitotomy Approach.

A modified lateral orbitotomy approach can also be considered in select cases, offering minimally invasive access to the mesial temporal lobe and avoiding the need for orbital retraction, as shown in the green triangle in the figure.^7^

### 2:47 Long Anatomical Axis of the Amygdala-Hippocampal Complex.

For lesions involving the mesial temporal lobe, the endoscopic transorbital approach can be considered an alternative to the aforementioned conventional approaches, especially for accessing its anterior and middle portions. The key concept behind this minimally invasive technique lies in the approach vector, which aligns with the long anatomical axis of the amygdala-hippocampal complex. This concept is similar to how this approach is used for trigeminal schwannomas involving the Meckel’s cave.

### 3:23 Transorbital Corridor.

This is a 3D model illustrating the transorbital corridor, lateral to the superior orbital fissure, for approaching the mesial temporal lobe. The yellow area represents the extent of the lateral orbitotomy, including the frontozygomatic suture, while the blue area shows the extent of the transorbital craniectomy. And this is a superior view of the transorbital pathway, primarily passing through the greater sphenoid wing. It’s important to note that the lesser sphenoid wing should also be removed when approaching the mesial temporal lobe.

### 3:56 Surgical Strategy for Mesial Temporal Lobe Glioma.

And this is our surgical strategy for the mesial temporal lobe glioma. The patient was positioned supine. A high-lying eyelid skin incision was made. Lateral orbitotomy and transorbital craniectomy were performed step by step. Piecemeal resection was carried out from anterior to posterior after opening the dura. Finally, reconstruction was done. Before demonstrating our video, we want to briefly outline the types of skin incisions and orbitotomies for the endoscopic transorbital approach.

### 4:30 Types of Skin Incisions for Endoscopic Transorbital Surgery.

There are three types of skin incisions: superior eyelid incision, high-lying eyelid incision, and infra-eyebrow incision. We prefer the first two incisions because they result in minimal scarring after surgery.

### 4:44 Types of Orbitotomies for Endoscopic Transorbital Surgery.

There are two types of orbitotomies that we can use for the transorbital approach.^8^ When orbitotomy is employed, it is called an extended endoscopic transorbital approach. In this case, we performed a lateral orbitotomy. This maneuver allows for a lateral-to-medial trajectory angle, which is why we always perform this when approaching the mesial temporal lobe.

### 5:06 Operating Room Setup.

Lastly, this illustration shows our OR setup for endoscopic transorbital surgery. Similar to endoscopic endonasal surgery, we always stand on the right side of the patient, regardless of a lesion’s laterality.

### 5:20 Intraoperative Photographs of Endoscopic Transorbital Surgery.

These are intraoperative photos of our transorbital surgery. We always use the endoscope holder to perform the endoscopic microsurgery using a bimanual technique.

### 5:35 Subperiosteal Dissection.

After making a high-lying eyelid incision on the right side, we detach the periorbita in a subperiosteal fashion from the lateral orbital wall, which consists of the zygomatic body and the greater sphenoid wing.

### 5:49 Drilling of Lateral Orbital Wall.

We’re then drilling the lateral orbital wall in an anteromedial direction using a high-speed drill, while protecting the periorbita with a silastic sheet.

### 5:59 Exposure of Deep Temporal Fascia.

Deep temporal fascia is exposed.

### 6:03 Lateral Orbitotomy.

Next, we perform a lateral orbitotomy. This step is essential for creating adequate working space. It allows us to achieve a lateral-to-medial working angle, which is a key step for accessing the mesial temporal lobe.

### 6:16 Transorbital Craniectomy.

We then drill the remaining greater sphenoid wing, known as the transorbital triangle, which is located between the superior orbital fissure and inferior orbital fissure. This step ultimately exposes the temporal dura.

### 6:30 Exposure of the Temporal Dura After Transorbital Craniectomy.

Here, you can see the temporal and frontal dura. The temporalis muscle is on your left. You can also see the lesser sphenoid wing at 2 o’clock, which we will also drill away. We are drilling the sagittal crest between the orbital apex and the temporal dura.

### 6:49 Flattening of Middle Fossa Floor.

We are now flattening the middle fossa floor, which also contributes to widening the narrow transorbital corridor.

### 6:56 Sagittal Crest.

Then we carefully fracture the sagittal crest.

### 7:01 Dural Opening.

Finally, after completing the transorbital craniectomy, including drilling away the lesser sphenoid wing, we open the dura from inferior to superior, up to the level of sylvian fissure.

### 7:13 Corticectomy.

We are performing a corticectomy under neuronavigation guidance in the direction of the temporal horn.

### 7:19 Removal of the Infiltrated Amygdala.

We are beginning to remove the tumor infiltrating the amygdala in a piecemeal fashion using suction, carefully discerning its consistency from normal brain tissue as we proceed along the axis of the amygdala-hippocampal complex.

### 7:34 Resection of the Tumor.

We are also using an ultrasonic aspirator to facilitate the resection of the tumor, which has a relatively friable consistency. It is worth noting that this 4K endoscope allows us to differentiate the tumor cells from normal brain tissue.

### 7:49 Subpial Resection.

In addition, the tumor was carefully removed in a subpial manner to protect the pial membrane on the medial margin of the uncus, as medial to this delicate membrane lies the crural cistern containing critical neurovascular structures.

### 8:04 Removal of the Infiltrated Hippocampus.

We are now removing the hippocampus infiltrated by the tumor.

### 8:08 Temporal Horn.

You can see the roof of the temporal horn coming into view above the hippocampus.

### 8:14 CSF Egress.

CSF is leaking from the temporal horn.

### 8:16 Hippocampus, Choroid Plexus, and Choroidal Fissure.

We are getting closer to the roof of the temporal horn, where you can see the choroid plexus extending along its roof. You can also see the remaining hippocampal head and body and choroidal fissure medial to the choroid plexus. It is crucial to dissect and remove the affected hippocampus below the level of the choroidal fissure because further dissection above this fissure could inadvertently damage the optic tract and thalamus.

### 8:44 Inspection of Resection Cavity.

We are inspecting the medial side of the resection cavity to check for any residual tumor. You can see the oculomotor-tentorial triangle from this transorbital view, including the ipsilateral optic tract coursing medially and the oculomotor nerve entering the roof of the cavernous sinus.

### 9:06 Final View of Endoscopic Transorbital Surgery.

This is the final view following resection through the extended transorbital corridor.

### 9:11 Reconstruction.

Reconstruction in transorbital surgery is very straightforward. After thorough hemostasis, an inlay DuraGen graft is placed.

### 9:21 Duraplasty.

We are then performing a watertight duraplasty without abdominal fat graft.

### 9:27 Postoperative 3D CT.

This is a postoperative 3D CT scan showing the extent of the transorbital craniectomy.

### 9:34 Postoperative MRI.

Postoperative MRI revealed a gross-total resection. The final pathology was glioblastoma.

### 9:41 Postoperative Outcome.

The patient tolerated the procedure well with no neurological deficits. He was discharged home on postoperative day 4. Later, he underwent concurrent chemoradiotherapy.

### 9:52 Conclusion.

In conclusion, compared to traditional transcranial approaches, the endoscopic transorbital approach provides straightforward access to the mesial temporal lobe, offering key advantages: no need for brain retraction, preservation of the lateral neocortex and optic radiations, minimal scarring compared to a standard craniotomy, and faster recovery due to its minimally invasive nature.

### 10:18 References.

Thank you.

## Disclosures

The authors report no conflict of interest concerning the materials or methods used in this study or the findings specified in this publication.

## Author Contributions

Primary surgeon: Hong. Assistant surgeon: Jeon. Editing and drafting the video and abstract: both authors. Critically revising the work: both authors. Reviewed submitted version of the work: both authors. Approved the final version of the work on behalf of both authors: Hong. Supervision: Hong.

## Supplemental Information

### Patient Informed Consent

The necessary patient informed consent was obtained in this study.
